# Patient Influencers: The Next Frontier in Direct-to-Consumer Pharmaceutical Marketing

**DOI:** 10.2196/29422

**Published:** 2022-03-01

**Authors:** Erin Willis, Marjorie Delbaere

**Affiliations:** 1 Department of Advertising, Public Relations, and Media Design University of Colorado Boulder Boulder, CO United States; 2 Edwards School of Business University of Saskatchewan Saskatoon, SK Canada

**Keywords:** social media, influencers, health, pharmaceutical marketing, direct-to-consumer advertising, relationship marketing, marketing, advertising, pharmaceuticals, ethics

## Abstract

Social media influencers are becoming an increasingly popular strategic communication tactic used across industry verticals, including entertainment, fashion, and beauty, to engage directly with consumers. Pharmaceutical companies have also recently entered the social media marketing arena and—within the bounds of governmental regulations—have found ways to build relationships directly with patients using covert persuasion tactics like partnering with social media influencers. Due to consumers’ negative perceptions of pharmaceutical companies, it makes sense that new marketing tactics are being used to establish and improve relationships with consumers. Previous research well documents the ethical dilemmas of direct-to-consumer advertising, and there is recent burgeoning literature on online covert marketing tactics. The academic and medical literature, however, is behind in regard to social media influencers used in health and medicine. This paper highlights and defines terms used in industry practice, and also calls for more investigation and sets forward a research agenda. As consumers spend more time online and patients continue to consult social media for health information, it is important that this new marketing trend does not go unnoticed.

## Background

Tasked with research and development, pharmaceutical companies aid in the worldwide prevention and treatment of illness and disease; these companies encounter fierce competition and (usually) work within the bounds of government regulations [1]. Pharmaceutical companies set aside a portion of their budgets for research and development but over time the amount spent on direct-to-consumer marketing has surpassed research and development [2]. Direct-to-consumer pharmaceutical marketing refers to the promotion of prescription medications to consumers as patients, instead of targeting only doctors [3]. Previous research purports that direct-to-consumer marketing is effective [4,5], especially in encouraging consumers to ask their doctors about specific medications. A survey by the Kaiser Family Foundation shows that after talking to their physician about a medicine they saw advertised, about 44% of patients who requested the advertised medication were ultimately prescribed this medication [6]. However, patient trust in the pharmaceutical industry is extremely low—only 58% of Americans trust pharmaceutical companies [7]. This poses a challenge for pharmaceutical companies and their direct-to-consumer marketing efforts.

Health care marketers are beginning to use the term patient influencer to refer to those who promote pharmaceutical medications and/or medical devices, allowing companies to “leverage the patient experience and expertise in the design, development and promotion of their products and services” [8]. Due to consumers’ negative perceptions of pharmaceutical companies, it makes sense that new tactics are being used to establish and improve relationships with consumers. A recent report by eMarketer noted that consumer response is highest when messages are delivered from social media influencers compared to brand-owned channels; further, content from influencers is more effective at meeting communication goals [9]. Pharmaceutical marketers’ entry into social media in general, and influencer marketing in particular, presents both opportunities and challenges relevant to the various stakeholders involved. Little published research is available in this area, despite the pharmaceutical industry’s increasing use of patient influencers. There are different forms of patient influence, and each involves patients in a different way. The purpose of this viewpoint paper then is to simply raise awareness of influencer marketing by pharmaceutical companies and generate research and debate on the use of influencers in health care.

The following sections of this paper will explore the concepts of expert patients, patient advocates, and digital opinion leaders. Expert patients are far removed from pharmaceutical companies; they exert influence on other patients via online health communities [10,11]. A patient advocate is also active online, but they influence other patients via their social media presence and raise awareness about a particular disease or illness [12,13]. Some patient advocates are directly involved in promoting pharmaceutical products. Some health care professionals are also digital opinion leaders whose influence is due to their active social media presence along with a reputation for being a leader in their field [14,15]. Table 1 summarizes these key concepts. Together, these different groups hold the potential to involve and influence patients in health and medical decision-making.

**Table 1:** Key concepts.

Concept	Explanation
Expert patient	Patients who are actively involved in online communities and who share disease experiences, information, and support with other patients and develop expertise in disease self-management.
Patient advocate	Third-party users of social media who are vocal in raising awareness of illness and disease. They are a specialized type of social media influencer and are recruited by pharmaceutical companies to participate in the development and promotion of pharmaceutical products.
Digital opinion leader	Health care professionals with an active social media presence who are seen as leaders among their peers.

## Online Communities and Expert Patients

Shared decision-making in health care requires collaboration between patients and physicians; in reality, however, there are many obstacles to patients collaborating with physicians, including technology barriers, health literacy levels, and access to health insurance [16]. Physicians’ time is also constrained; a recent survey reported that 51% of physicians spend 9-16 minutes with patients [17], which could have an impact on health outcomes [18-20]. Patients then often turn to the internet and social media to fill the gaps in the health care system.

Online communities can be a source of health information and peer empowerment for patients. Online communities are social platforms where “people come together to get and give information or support, or learn or to find company” [21]. A study of an online community for patients with arthritis found that members developed expertise in disease self-management in part by modeling behaviors that were discussed in the community [22]. By sharing disease experience, patients are able to “crowdsource” answers to their current situation, and “see” the outcome of different strategies through the shared user-generated content [23]. A study by Fox, Ward, and O’Rourke [24] found evidence to suggest that online communities result in the development of *expert patients*. The researchers found that active participants ultimately became expert patients in the online community; they shared information within the community and received valuable support from other patients. Although these patients became experts, their expertise was limited to the biomedical model that supports pharmaceutical interventions. They did not become experts in a wide range of approaches to weight loss [24]. Despite this limitation, the authors suggest that informed patients are in turn informed consumers who are engaging directly with providers on health technologies as opposed to only indirectly through a medical professional.

Research found that patients often join online communities in search of information about disease treatment options, including pharmaceutical medications [25-27]. For instance, a recent content analysis (N=1960) of 4 online health communities found that medication (N=568) is one of the most popular topics discussed by members [28]; medication adherence is discussed in terms of the presence of disease symptoms, and members share their experiences with specific medications, focusing on the specific barriers to adherence (eg, cost, side effects) [29]. The number of online health communities has rapidly increased as more patients desire to access alternate sources of information as well as connect with other patients with the same illness or disease [30,31]. Although previous research notes that online health education programs about chronic disease self-management positively impact participants’ health outcomes [32,33], scant research studies the health effects of online peer-to-peer communities and the influence of user-generated content on health attitudes and behaviors.

## Social Media Influencers

Recent marketing efforts by pharmaceutical companies have focused on digital advertising and engagement tactics to connect with patients and build relationships [34]. In fact, pharmaceutical companies are using digital data analytics to formulate target audience profiles and direct-to-consumer marketing strategies that take a holistic approach and consider patients’ overall lifestyle preferences and their well-being, not only the disease diagnosis [35]. This new focus describes pharmaceutical companies’ efforts in relationship marketing, using social platforms to speak with patients and learn about their preferences [36]. Relationship marketing draws from the practice of public relations and attempts to foster customer loyalty and long-term retention.

The pop culture term *influencer* refers to a brand’s commercialization of the relationship between an influential social media user and his or her followers [37]. Social media influencers can be defined as “third-party users of social media who have achieved micro-celebrity status in the form of large followings on social media platforms and who have a position of influence on their audience” [38]. From a strategic communication perspective, social media influencers are actors who influence “organizational stakeholders through content production, content distribution, interaction, and personal appearance on the social web” [39]. The relationship between an influencer and his or her followers is fundamental to a brand’s success because it should drive positive word-of-mouth and purchase intentions [40]. eMarketer reported that more than two-thirds of North American retailers use some form of influencer marketing [41]. Brands are not just selling products but entire lifestyles, employing social media influencers to build trust through authentic, curated content. Followers perceive influencers as being knowledgeable experts on specific topics, with relevant sources for information and support for their opinions and behaviors [42].

Influencers may or may not have demographics and psychographics that are similar to those of the consumer, but they are similar to the consumer in that they share a common interest in the topic of the group [43]. Influencers are chosen to represent a brand for a number of reasons, including industry popularity [44], quality of social media content [45], experience with brand partnerships [46], and audience demographics [47]. Choosing an influencer is part of a larger marketing strategy that usually supports campaign goals and broader communication objectives [48].

## Pharmaceutical Influencers: Patients, Advocates, and Opinion Leaders

In direct-to-consumer pharmaceutical marketing, it can be risky for brands to activate paid social media influencers for a number of reasons: navigating Food and Drug Administration (FDA) and Federal Trade Commission (FTC) regulations, concerns about authenticity, and managing consumer engagement [49-52]. Kim Kardashian’s endorsement of Diclegis, a medication to treat morning sickness, is a prime example of the risks inherent in celebrity influencers partnering with pharmaceutical companies. In 2015, Kardashian and Duchesnay, the drug manufacturer, were found to have violated FDA regulations by not properly disclosing the risks and side effects of the drug in Kardashian’s Instagram post about how the drug helped her combat morning sickness during her pregnancy [53,54]. Rather than continuing to pursue partnerships with celebrity influencers, pharmaceutical marketers have instead turned to health and medical opinion leaders as well as *patient advocates*. Typically, patient advocates are active on social media, tend to raise awareness of illness and disease, and are considered micro- or nano-influencers. These types of influencers typically have a smaller number of followers but cultivate more targeted communities, generating higher engagement rates and building stronger relationships with stakeholders than celebrity influencers [55]. Pharmaceutical brands are beginning to opt for micro- and nano-influencers who have very specific audiences that may be primed for health messaging. A recent article in Vox described the value of patients to pharmaceutical marketers in building brands [56]. Lived experience is something that patients have in common with each other, and cannot be replicated; thus, patient influencers are simply commercializing their lived health experience. Patient influencers used in this way attempt to create an emotional linkage with followers by sharing strategic and curated pieces of their illness and disease experience.

According to a recent comprehensive review of medical marketing expenditures in the United States, pharmaceutical companies spend nearly 70% of their promotional budgets marketing to health care professionals, or just over US $20 billion in 2016 [57]. These marketing activities include prescriber detailing, free samples, direct physician payments, and disease education. A substantial portion of the direct payments goes toward sponsoring key opinion leaders [58]. Physicians have long been influencing other physicians by giving keynote speeches and lectures at educational events and serving as product champions or product endorsers for pharmaceutical companies [59]. The industry now also recruits physicians who are digital opinion leaders; that is, health care professionals with an active social media presence who are seen as leaders among their peers, but who might have different characteristics than traditional key opinion leaders [58]. Pharmaceutical marketers are beginning to use the term *influencer* to describe this type of opinion leader. Online marketing services like Klear compile lists of the top physician influencers across social media platforms [60].

## Blurred Lines: Patient Empowerment or Patient Deception?

Noticeably absent from the literature is the role advertising and marketing agencies play in relationship marketing and the use of influencers to promote pharmaceutical brands. Patient advocates self-select to engage in pharmaceutical marketing efforts and are supplied with promotional collateral to share with their followers so that their persuasive messaging stays within the boundaries of disease awareness—the type of direct-to-consumer advertising permitted by regulating government agencies. However, little is published about this phenomenon. Sites like WEGO Health are actively recruiting patients as social media influencers or patient advocates so as to connect pharmaceutical companies and other health and medical brands with patients to commercialize their lived disease experience [8].

## Research Agenda and Conclusion

### Overview

No known academic research is published on pharmaceutical influencer marketing. There are some industry papers on the topic [61-63]. Although industry practice moves quickly, most details of the relationship between pharmaceutical companies and advertising agencies or digital marketing companies are protected by intellectual property laws and nondisclosure agreements. Our purpose here is to suggest a research agenda. We argue that it is a distinct possibility that pharmaceutical companies could look next to online patient communities. What would happen if they partnered with online communities already successful at encouraging patient expertise? Although marketers might argue that this type of direct-to-consumer marketing is beneficial to creating empowered and informed patients, more research needs to be done to better understand this increasingly popular industry practice. Investigation needs to be conducted in the areas of patient influencers and patient advocates on social media and how these opinion leaders are being used in health care marketing.

To advance knowledge in this area, we propose the following research questions for investigation and intellectual debate.

### Research Question 1: Are the Ethical Issues the Same for Influencer Marketing as Traditional Direct-to-Consumer Advertising Channels?

The debate on the risks and benefits of pharmaceutical direct-to-consumer advertising is complex; it is worth noting, however, that various studies of online drug advertisements have shown that in some cases suspect claims are made and in general the ads tend to overemphasize the benefits of the drug [64,65]. Direct-to-consumer advertising may also create a preference among consumers for recently launched pharmaceutical drugs over more established treatments because of heavy advertising campaigns; this can lead the market to concentrate only on newer products, excluding older, traditional options [66,67].

When pharmaceutical companies recruit patient advocates as influencers, they are in essence adapting the opinion leader strategy that has proved very successful for them with physicians to a new target market, much the same way they did with direct-to-consumer advertising. Similar concerns could also be raised with the practice of targeting consumers with a new type of marketing strategy as opposed to targeting physicians. Physicians possess many years of medical education and training that afford them expert knowledge when they are the target of persuasion tactics. They can draw on their high levels of topic knowledge in addition to their knowledge of persuasion when coping with persuasion attempts (ie, the attempts of another physician to influence their attitudes about a pharmaceutical brand). Consumers, on the other hand, do not have this same level of medical expertise and instead must rely on their knowledge of persuasion when assessing the validity of a persuasion agent’s claims [68].

### Research Question 2: How Does the Influence of Patient Experts, Influencers, Celebrity Patients, and Small Patient Forums Differ From More Traditional Advertising Venues?

Future research should explore whether the new modalities enhance the credibility of messaging within a larger societal context characterized by celebrity culture, mistrust of formal institutions, and the more recent and profound social polarizations.

Influencer marketing shares similar characteristics to native advertising, including promotion that does not hint at advertising as well as not using highly persuasive messaging [69-71]. The interests of consumers are the primary consideration in native advertising, not only the brand; thus, content is usually relevant and useful to audiences [72]. The blurring of boundaries between paid and earned content and the prevalence of covert persuasion attempts raises questions of transparency, ethics, and trust, which has been the focus of native advertising research [73,74]. Online communities and social networks encourage the exchange of health care information, including symptoms, treatment options, and potential side effects, and opinions about experiences with doctors and other health care providers [75]. This online exchange of health care information has the potential to influence health care decision-making [76]. The potential impact of covert marketing tactics on health care decision-making is not yet known.

Beyond marketing strategy, we need to understand how this type of influence affects medical decision-making and what effect that has on patients’ health outcomes. To what extent do influencers affect decision-making by providers and patients? To what extent have these decisions caused harm (or good)? What factors affect whether the consumer will follow the influencer or their shared recommendations? As the media environment continues to fracture and consumers’ trust in government offices, pharmaceutical companies, and health care professionals declines, employing patient influencers may be a way to rebuild that trust while also improving consumers’ attitudes and behaviors related to health [77-79]. Research shows that social media influencers have a high return on investment [80], but what does that mean for pharmaceutical companies and patients’ health outcomes?

### Research Question 3: Who Are the Patient Influencers? Who Is Recruited by Pharmaceutical Companies and Who Approaches Pharmaceutical Companies With Intent of Monetizing a Medical Condition? Who Receives Compensation, and Who Does Not?

As mentioned previously, micro- or nano-celebrities are beginning to act as patient influencers for pharmaceutical marketers. In addition to this type of celebrity, patient advocates are also playing the role of patient influencer. Future research should explore whether there are differences in the conditions surrounding the use of each type of patient influencer and whether there are differences in type or level of compensation. Additionally, future studies should explore the motivation of these different patients to act as influencers; specifically, whether some could be considered market mavens as opposed to opinion leaders. A market maven is someone with generalized market knowledge, across multiple product categories, who enjoys helping other consumers make better marketplace decisions [81]. The concept of a market maven is related to, but distinct from, that of an opinion leader. A study that investigated an online community of ecstasy users suggests that this last characteristic is a key differentiator between market mavens and opinion leaders [81]. Opinion leaders’ interactions with others are motivated by self-involvement, while market mavens’ interactions with others are motivated by a desire to help others. According to O’Sullivan [82], market mavens are individuals who share marketplace information with the goal of reducing consumption-related risks of other consumers, essentially filling information gaps that exist within a market. It is our contention that the concept of a marketplace can be extended to the domain of chronic disease, in which people are consuming medications, health care aids, and perhaps most importantly, information related to their disease. We believe that the concept of market maven holds great potential for improving our understanding of the role that online health communities play in consumers’ health decisions.

### Research Question 4: What Is the Potential for Misinformation in Influencer Marketing? Who Is Responsible for Harm Caused in These Forums of Misinformation?

For decades, pharmaceutical companies have modified direct-to-consumer marketing strategies, being early adopters of communication platforms [83]. Burgeoning research explores pharmaceutical companies’ use of branded and unbranded content on social media, including influencer marketing campaigns [84,85]. In November 2019, the FTC updated their social media marketing guidelines on how brands and influencers should work together and what is necessary to disclose [51]. However, the FTC’s guidelines are vague and up to interpretation if no pharmaceutical brand name is mentioned. So far, these campaigns appear to be limited to partnering with existing influencers to promote products.

A recent study of the general population found that social influencers impacted diet-related decisions for 32% of survey respondents [86]. The authors of the study expressed concern that in spite of this impact, most social influencers have no official qualifications as dietitians or nutritionists and frequently share information without any scientific evidence [86]. However, many of these influencers share from their own personal experience and that brings a certain type of value all its own to patients. Although this type of influence is frustrating to health care professionals, it is fascinating to marketers. People are persuaded by powerful personal stories in the absence of “hard” evidence. As more consumers turn to the internet as a primary source for health information, consideration needs to be given to how this information affects consumers’ decision-making. Studies have shown that consumers can be skeptical about their doctor’s motives for prescribing brand name drugs when the brand’s logo appears on objects in the doctor’s office [87]. Preliminary work investigating consumer skepticism with online forums managed by pharmaceutical brands suggests that consumers are less skeptical about online communication from pharmaceutical companies and do not recognize these sponsored communities as persuasion attempts [88].

As direct-to-consumer advertising continues to evolve and innovate, the use of influencer marketing will also continue to increase; this phenomenon needs more investigation as consumers spend an increasing amount of time online and engaging with social media, and as pharmaceutical companies collect more information and connect directly with patients.

## Abbreviations

FDA: Food and Drug Administration
FTC: Federal Trade Commission
